# Antioxidant and tyrosinase inhibitory activities of traditional fermented *Rosa* from Dali Bai communities, Northwest Yunnan, China

**DOI:** 10.1038/s41598-021-02160-y

**Published:** 2021-11-22

**Authors:** Bayi Lang, Yanqiang Zhao, Rong Yang, Aizhong Liu, Sailesh Ranjitkar, Lixin Yang

**Affiliations:** 1 Bio-Innovation Center of DR PLANT, Kunming Institute of Botany, Chinese Academy of Sciences, Kunming, 650201 Yunnan China; 2 Key Laboratory of Economic Plants and Biotechnology, Kunming Institute of Botany, Chinese Academy of Sciences, Kunming, 650201 Yunnan China; 3 Center of Biodiversity and Indigenous Knowledge, Kunming, 650034 Yunnan China; 4 College of Forestry and Vocational Technology in Yunnan, Kunming, 650224 Yunnan China; 5 Southwest Forestry University, Kunming, 650224 Yunnan China; 6 N.Gene Solution of Natural Innovation, Kathmandu, GPO, 44614 Nepal; 7 Faculty of Humanities and Social Science, Mid-Western University, Naya Bato, Lalitpur, 44600 Nepal

**Keywords:** Biological techniques, Microbiology techniques, Plant sciences

## Abstract

Traditional fermented *Rosa* (TFR) is a typical food and medical product among the Dali Bai people, and its popularity is growing. A few studies have looked into TFR's medicinal advantages, linked germplasm resources, traditional processing procedures, and functional food qualities. Our goal was to look into *Rosa*'s traditional processing, examine the dominant strains in TFR, and prove how these strains affected antioxidant and tyrosinase inhibitory activities. We used a snowball selection strategy to pick 371 informants for a semi-structured interview, supplemented with direct observations and sample collection. A microbial strain was isolated and identified from a TFR sample collected in the field. We synthesized TFR in the lab using the traditional way. Both of 2, 2-diphenyl-1 picrylhydrazyl (DPPH) free radical scavenging and tyrosinase inhibitory properties of the fermented solution of *Rosa* 'Dianhong' have been tested in this study. Altogether 15 species belonging to the genus *Rosa*, which are utilized in herbal medicine and fermented foods. *Rosa* 'Dianhong' was the Bai community's principal species with considerable cultural value and consumption. Raw *Rosa* petals included 15 major flavonoids and phenols, which were identified as TFR's active components. TFR-1 was discovered to be the dominating microbial strain in TFR, increasing total phenolic and flavonoid content in the fermented solution of *Rosa* 'Dianhong' by 0.45 mg GAE/ml and 0.60 mg RE/ml, respectively, after 30 days. TFR-1 also exhibited promising activity in terms of DPPH free radical scavenging and tyrosinase inhibition. TFR showed potent antioxidant and free-radical scavenger properties and is beneficial in skincare and nutrition, according to the findings. TFR's medicinal and edible properties suggest that it could be used as a cosmetic or nutraceutical product.

## Introduction

Fermentation has a long history in human food production and is a valuable process in food industries, making food products available throughout the year^1,2^. Fermentation of edible items improves product quality and creates diverse flavor components, which boosts consumer acceptability^3–5^. Furthermore, by adding salt and producing acid and ethanol, the fermentation process can improve the nutritional and functional qualities of foods while also extend their shelf life^2^. Microorganisms and their enzymes cause fermentation, which is a biochemical alteration of the fundamental food matrix. Traditional knowledge of the fermentation of edibles has shown pharmacological activity that can be used to cure a variety of diseases. Fermentation, for example, allows for the manufacture of broccoli products that are safe, stable, and high in sulforaphane. These fermented foods are fantastic dietary supplements^6^. It also contains anti-cancer^7,8^, anti-diabetes^9^, and anti-obesity^10^ properties, as well as helping to alleviate behavioral issues linked with autism spectrum disorder^11^. Fermentation also enhances the antioxidant activity and tyrosinase inhibitory activity of phenols^12^ and flavonoid antioxidants^13^.

The adaptive nature of the fermentation process within a given region arises from centuries of human relationships with the microbial community in the environment. Microorganisms, inorganic components, and their interactions all play a role in fermentation processes and product creation^14^. In traditional fermentation processes, such as those of *Brassica oleracea* L., *Oryza sativa* L., and *Glycine max* (Linn.) Merr., microbial sources are either internal to plants or derived from external surfaces and the surrounding environment. Fungi and bacteria, particularly yeast and lactic acid bacteria populations among the microorganisms that increase the quality of traditional fermentation products^3,4^. Traditional fermentation is a promising method for isolating certain microorganisms’ impact on biosynthesis or breakdown of bioactive substances.

Traditional fermented *Rosa* (TFR) is a biocultural heritage among Dali Bai communities in northwest Yunnan, China, which has evolved over a long time as a result of interactions between local cultures and their environment^15^. TFR is widely acknowledged in Dlai Bai communities as a medical food that nourishes the body and keeps the skin smooth. However, until today, knowledge of traditional TFR processing has been preserved within the community. TFR’s plant resources, fermentation method, and bioactivity have never been comprehensively described. When the globe is confronting a pandemic like Covid-19, research into traditional knowledge around such a vital food source and its bioactivity is critical. Functional foods with health advantages are in high demand. To that end, the goal of this study was to determine the main microbial strains and relative bioactivities of TFR, as well as clarify the plant resources and conventional processing methods employed in TFR. Our research not only contributes to biocultural preservation, but it also gives critical information for the future development of TFR-based pharmaceuticals and prospective nutraceuticals.

## Results

### Diversity of the genus of *Rosa* in Dali Bai communities

Our informants told the interviewers about 15 *Rosa* species used in Dali for traditional applications. Both wild and cultivated species are in use as food and medicine. Table 1 shows the usage information for these 15 species. The informants reported that *Rosa* species have anti-aging properties; cure rheumatism and dehydration; activate blood circulation; and possess detoxification, insecticidal, and diuretic properties. These plants are used as medicine, food, and fragrance, with different methods and frequency of use.

**Table 1:** Summary of traditional applications of the genus *Rosa* in Dali Bai communities.

Scientific name	Chinese names with Pinyin	Parts used	Processing methods	Usage and efficacy	Geographical distribution	*ICF*	*f*
*Rosa* ‘Dianhong’	滇红玫瑰 Dianhong	Petal	Fresh petals fermented with sugar and a little wine or air-dried	Food; petal fermentations are hypoglycemic and hypolipidemic, maintain health and smooth skin, reduce weight, promote blood circulation, protect liver, and nourish stomach; tea and wine of petals eliminate breast lumps	Cultivated in northwest Yunnan	0.93	0.99
*Rosa damascena* Mill	大马士革玫瑰 Damashige	Petal	Extracted using water	Extracting essential oil and adding to cosmetics	Cultivated in northwest Yunnan	0.91	0.96
*Rosa laevigata* Mich	金樱子 Jinyingzi	Leaf, root, and fruit	Air-dried	Fruit for syrup or wine with anti-aging functions; root for medicine for curing rheumatism, dehydration, activation of blood circulation, detoxification, and insecticidal treatment; external use of leaves against sores, burns, and scalds	Distributed on sunny hillsides and in field-side and stream-side shrubbery at elevations of 200–1400 m	0.79	0.69
*Rosa* ‘Mohong’	墨红玫瑰 Mohong	Petal	Fresh petals fermented with sugar and a little wine or air-dried	Food, for nourishing the body and keeping skin smooth	Cultivated in northwest Yunnan	0.77	0.37
*Rosa rugosa* Thunb	玫瑰 Meigui	Bud	Air-dried	Food and medicine, for promoting qi flow to relieve depression, harmonizing blood, and relieving pains; for treating hematogenous gastralgia, menoxenia, and traumatic injury	Cultivated in northwest Yunnan	0.67	0.54
*Rosa chinensis* Jacq	月季 Yueji	Root, stem, and bud	Air-dried	Buds for treating menoxenia, amenorrhea, dysmenorrhea, pains, and swelling from static blood, traumatic injury, and scrofula; root and stem for traumatic injury, irregular menstrual bleeding, and spermatorrhea; external use against pains and swelling from furuncles	Distributed at forest edges or in shrubbery at elevations of 800–2600 m	0.62	0.46
*Rosa banksiae* Ait. var*. normalis* Regel	单瓣白木香 (变种) Danbanbaimuxiang	Root and root bark	Minced, steamed, and dried	Medicine, for promoting blood circulation, regulating menstruation, and detumescence	Distributed in valleys and ditches at elevations of 1600–2500 m	0.56	0.69
*Rosa murielae* Rehd. et Wils	西南蔷薇 Xinanqiangwei	Fruit	Air-dried	Medicine, for promoting blood circulation, dispersing blood stasis, diuresis, tonifying the kidney, and relieving cough	Distributed in shrubbery on hillsides or gully sides at elevations of 1800–2400 m	0.53	0.31
*Rosa macrophylla* Lind	大叶蔷薇 Dayeqiangwei	Fruit	Air-dried	Medicine, for promoting blood circulation, dispersing blood stasis, diuresis, tonifying the kidney, and relieving cough	Distributed in shrubbery at elevations of 2700–3600 m	0.49	0.36
*Rosa roxburghii* Tratt. f. *normalis* Rehd. et Wils	单瓣缫丝花 Danbansaosihua	Fruit	Refined with sugar or used for making wine	Food and medicine, also as an ingredient in syrups and wines, with an anti-aging function	Distributed in hillside shrubbery at elevations of 500–2500 m	0.43	0.56
*Rosa banksiae* Ait	木香花 Muxianghua	Root and petal	Rhizomes minced, steamed, and dried; petals air-dried	Root for medicine, with anti-dysentery and hemostatic functions; petals for essential oil	Distributed in roadside shrubbery at elevations of 1500–2650 m	0.43	0.63
*Rosa sertata* Rolfe	钝叶蔷薇 Dunyeqiangwei	Fruit	Air-dried	Medicine, for promoting blood circulation, dispersing blood stasis, diuresis, tonifying the kidney, and relieving cough	Distributed on hillsides or in sparse forest at elevations of 1750–3950 m	0.41	0.26
*Rosa gallica* L	法国蔷薇 Faguoqiangwei	Petal	Fresh petals fermented with sugar and a little wine or air-dried	Functional food for maintaining health and smooth skin	Cultivated in northwest Yunnan	0.36	0.12
*Rosa odorata* (Andr.) Sweet	香水月季 Xiangshuiyueji	Root and bark	Minced, steamed, and dried	For treating carbuncles, furuncles, and ulceration	Distributed at edges of hillside forests or in roadside shrubbery at elevations of 700–3300 m	0.28	0.17
*Rosa centifolia* L	百叶蔷薇 Baiyeqiangwei	Petal	Used fresh or air-dried	Functional food for maintaining health and smooth skin	Cultivated in northwest Yunnan	0.11	0.08

The Dali Bai communities primarily use 10 of the 15 species for medicine, 7 for food, and 2 for fragrance. *Rosa damascena* is one of the main species used for essential oil extraction. Of the seven edible species, *Rosa* ‘Dianhong’ scored the highest informant consensus factor *(ICF)*^16^ and use frequency *(f)*^17^ values, indicating its cultural value and extent of consumption (Table 1). *Rosa* ‘Dianhong’ is used in petal tea, wine, sugar, and TFR. TFR is the most popular among these uses.

### The TFR in Dali Bai communities

**Figure 1:**
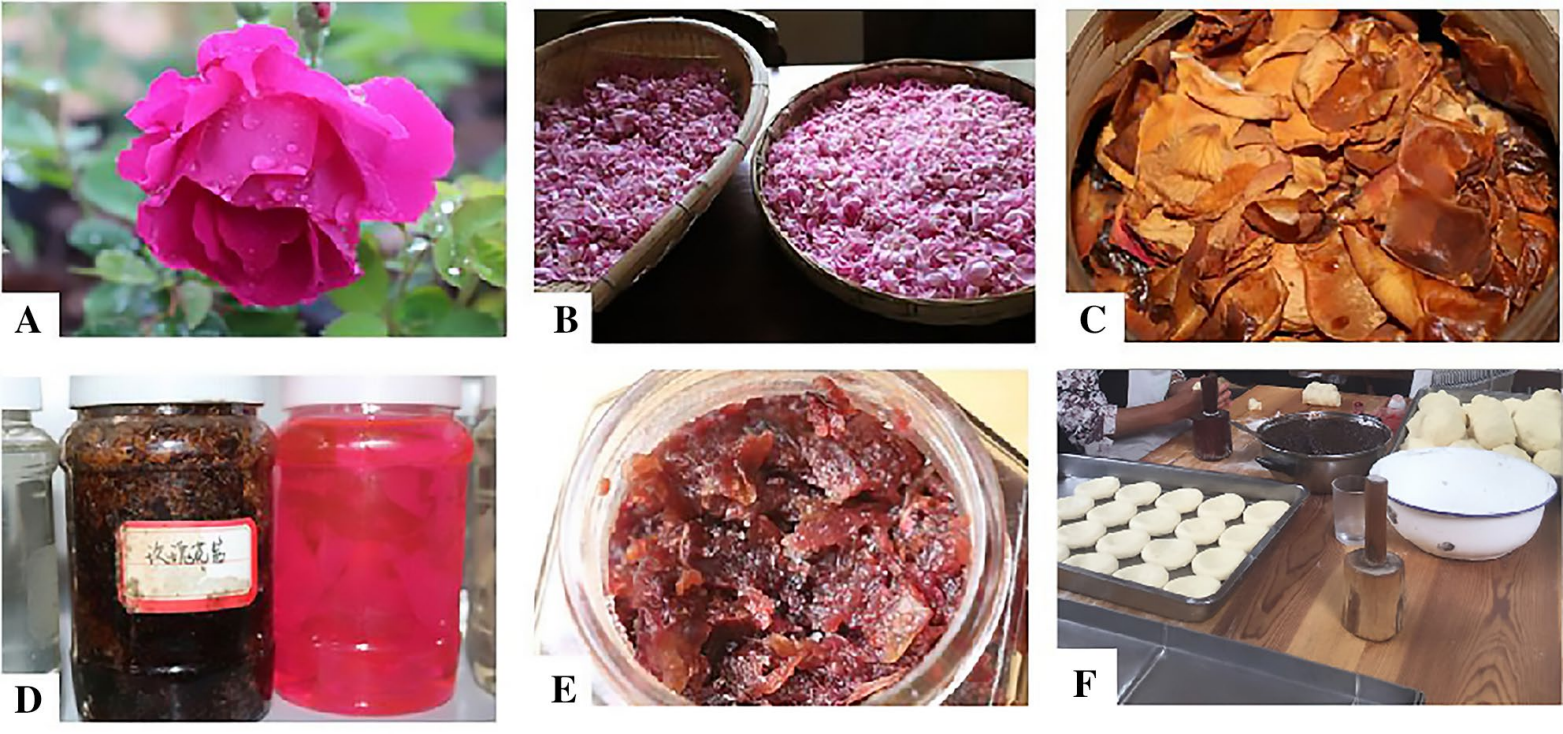
Traditional uses of *Rosa* by Bai people in Dali, northwest Yunnan: (**A**) *Rosa.* ‘Dianhong’. (**B**) Drying fresh *Rosa* petals to remove rain and dew. (**C**, **D** and **E**) Traditional fermented *Rosa* and (**F**) Cake workshop of traditional fermented *Rosa* in Heqing, Dali.

TFR in Dali was prepared by natural fermentation. Petals picked from locally available edible *Rosa* plants were the main ingredient in TFR (Fig. 1). The traditional planting of edible *Rosa* resources including *Rosa* ‘Dianhong’, *Rosa* ‘Mohong’, *Rosa laevigata*, *Rosa rugosa*, *Rosa roxburghii*, *Rosa gallica*, and *Rosa centifolia. Rosa* ‘Dianhong’ was mainly used in TFR preparations, while *Rosa* ‘Mohong’ was used as an ingredient of TFR only in Heqing county. According to our informants, the thinner petals of *Rosa* ‘Dianhong’ have better flavor after fermentation compared with those of *Rosa* ‘Mohong’.

All informants mentioned that TFR benefits the skin. The majority of informants (83.3%) told us that TFR can nourish the stomach, and a few informants mentioned that TFR is hepatoprotective and refreshing. Although none of the informants could explain the pharmacological reasons, these traditional uses hint at potential therapeutic value.

### Microbial strain identification

Microbial strains were isolated from TFR prepared traditionally by the Bai people in Dali. The ITS fragment from the dominant strain, TFR-1 (Table 2), shared 99.84% similarity with that of *Saccharomyces rouxii*. Therefore, the strain TFR-1 was named *Saccharomyces rouxii* TFR-1. The strain is now preserved in the China General Microbial Culture Collection Center (CGMCC) with the sample number CGMCC19335.

**Table 2:** ITS fragment from TFR-1.

Microbial name	ITS fragment
TFR-1	tcgtaacaag gtttccgtag gtgaacctgc ggaaggatca ttatagaaaa tgacgtgaac tcttaacgga gttctctcaa agtgttggag gggaaggcct gcgcttaatt gcgcggctgt ttttaatctc ctccgccttt gatacacaca ttggagtttc tacttttttg ttctctttgg gagggttctg ctctcccaga ggtaaacaca aacaatcttt tattatacta ttaacacagt caaatgaatt ttaaaaacaa aatattcaaa actttcaaca acggatctct tggttctcgc atcgatgaag aacgcagcga actgcgatac gtaatgtgaa ttgcagaatt ccgtgaatca tcgaatcttt gaacgcacat tgcgcccctt ggtattccgg ggggcatgcc tgtttgagcg tcatttccct ctcaaacttt acgtttggta gtgagcgata ctctactctg gagtttgctt gaaaatggga ggccataggc gaagcattgc tttccaatcc tgcggccctc tgcttacttc cccttgtggg ttgtggcagg ggaaagcggg aggcgccttg ccacgatagt cgtattaggt tttaccgact cggcgaaagt gaagaggttt gctttttaaa aagaagcagg cagcgtctgg cttgacaaaa ttctcaaagt ttgacctcaa atcaggtagg attacccgct gaacttaagc atat

### Pharmacological properties of FSR

#### Antioxidant activity

**Figure 2:**
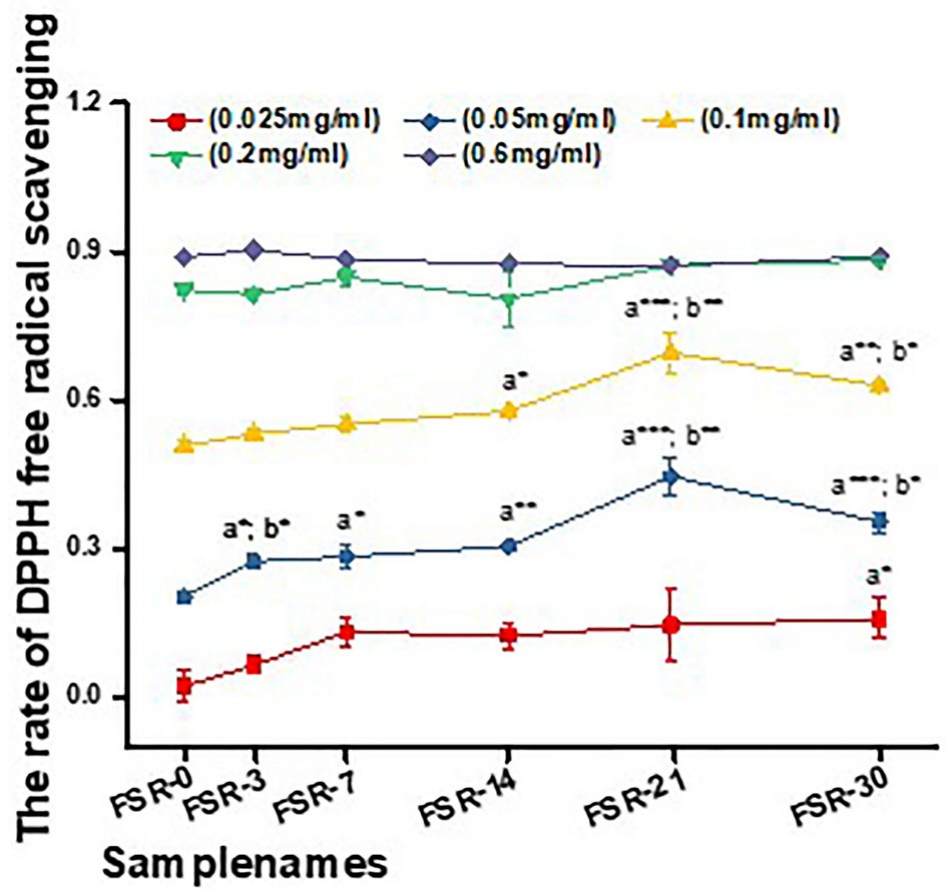
Effects of fermentation time and concentration of FSR on DPPH radical scavenging activity. Each value represents the mean ± SD (*n* = 3). Different numbers of asterisks represent different levels of significance from one-way ANOVA: a, compared with FSR-0; b, compared with the previous sample; *P < 0.05; **P < 0.01; ***P < 0.001.

The free radical scavenging activity of FSR after different periods of fermentation were examined. As shown in Fig. 2. At low concentration (i.e., 0.025 mg/ml, 0.05 mg/ml, and 0.1 mg/ml), samples FSR-3, FSR-7, FSR-14, FSR-21, and FSR-30 showed better DPPH free scavenging activities than FSR-0. At a concentration of 0.05 mg/ml, this difference was significant (*P* < 0.05). FSR-21 had the highest DPPH scavenging activity, reaching 44.60%. At concentrations greater than 0.2 mg/ml, there was no significant difference in the DPPH scavenging activity of FSR-3, FSR-7, FSR-14, FSR-21, and FSR-30 compared with that of FSR-0. However, with increasing concentration, the trend for greater scavenging activity with longer fermentation began to flatten. DPPH radical scavenging rates of these samples were higher than 85% after 30 days. Table 3 shows the IC_50_ values for FSR and the positive control.

**Table 3:** IC_50_ values for DPPH radical scavenging by fermentation solution of *Rosa* ‘Dianhong’ (FSR).

Samples	IC_50_ (mg/ml)	Gallic acid (IC_50_, μg/ml)
FSR-0	0.105	8.711
FSR-3	0.096	
FSR-7	0.088	
FSR-14	0.089	
FSR-21	0.065	
FSR-30	0.073	

#### Tyrosinase inhibition activity

**Figure 3:**
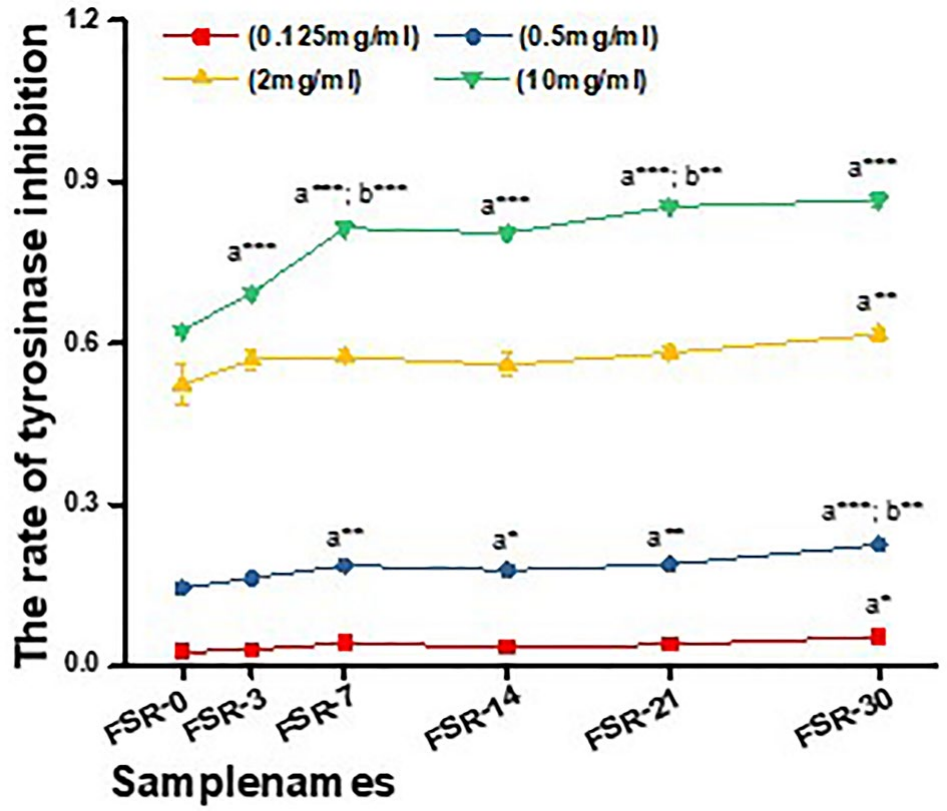
Effects of fermentation time and concentration of fermentation solution of *Rosa* ‘Dianhong’ (FSR) on tyrosinase inhibition. Each value represents mean ± SD (*n* = 3). Different numbers of asterisks represent different levels of significance from one-way ANOVA: a, compared with FSR-0; b, compared with the previous sample; *P < 0.05; **P < 0.01; ***P < 0.001.

At a concentration of 0.125 mg/ml, tyrosinase inhibition activity of FSR was insignificant at all time points compared with FSR-0, except in the FSR-30 sample (5.48%; *P* < 0.05) (Fig. 3). However, as the concentration of FSR increased, tyrosinase inhibition activity also increased. At a concentration of 0.5 mg/ml, tyrosinase inhibition by FSR-7, FSR-14, FSR-21, and FSR-30 was significantly different from that by FSR-0 (*P* < 0.05). The confidence level for the FSR-30 sample was higher (*P* < 0.001), and FSR-30 was also significantly different from the FSR-21 sample (*P* < 0.05). Although only the inhibitory activity of FSR-30 showed a statistical difference compared with FSR-0 at a concentration of 2 mg/ml, the mean value of tyrosinase inhibition increased with increasing fermentation time. At a concentration of 10 mg/ml, tyrosinase inhibition by FSR-3, FSR-7, FSR-14, FSR-21, and FSR-30 was significantly higher than that of FSR-0 (*P* < 0.001). The highest inhibition rate was 86.58% and was for FSR-30. Among all samples, longer FSR fermentation duration resulted in greater inhibition of tyrosinase activity. Table 4 shows the IC_50_ values for FSR and the positive control.

**Table 4:** IC_50_ values for tyrosinase inhibition by fermentation solution of *Rosa* ‘Dianhong’ (FSR).

Samples	IC_50_ (mg/ml)	Kojic acid (IC_50_, μg/ml)
FSR-0	3.508	4.986
FSR-3	2.618	
FSR-7	1.901	
FSR-14	2.033	
FSR-21	1.734	
FSR-30	1.494	

#### Nutrients in FSR and phytochemical profile

The total phenolic contents of FSR-3, FSR-7, FSR-14, FSR-21, and FSR-30 were significantly higher than that of FSR-0 (*P* < 0.001) (Table 5). Longer duration of fermentation increased the total phenolic contents of FSR. However, none of the differences between adjacent fermentation intervals, FSR-3, FSR-7, FSR-14, FSR-21, and FSR-30, were significant.

**Table 5:** Total phenolic contents and total flavonoid contents in fermentation solution of *Rosa* ‘Dianhong’ (FSR).

Samples	Contents	
	Total phenols (mg GAE/ml)	Total flavonoids (mg RE/ml)
FSR-0	2.5371 ± 0.0303	3.8109 ± 0.0218
FSR-3	2.8851 ± 0.0439^a^***	3.9182 ± 0.0126
FSR-7	2.9118 ± 0.0366^a^***	4.0791 ± 0.0769^a^**^; b^*
FSR-14	2.8971 ± 0.0349^a^***	4.0102 ± 0.0507^a^**
FSR-21	2.9641 ± 0.0377^a^***	4.1826 ± 0.0249^a^***^; b^*
FSR-30	2.9861 ± 0.0239^a^***	4.4105 ± 0.0221^a^***^; b^**

Values are shown as means ± SD (*n* = 3). Different numbers of asterisks represent different levels of significance from one-way ANOVA: ^a^compared with FSR-0, ^b^compared with the previous sample; *P < 0.05; **P < 0.01; ***P < 0.001. GAE, gallic acid equivalents; RE, rutin equivalents.

FSR-7, FSR-14, FSR-21, and FSR-30 showed a significant difference in total flavonoid content (*P* < 0.01) compared with FSR-0 (Table 5). However, different from the total phenolic contents, across the whole fermentation process, the values for total flavonoid contents of FSR-7, FSR-21, and FSR-30 were significantly different from those at the previous time point (*P* < 0.05). This indicated that fermentation duration plays an important role in increasing total flavonoid content in FSR.

#### Correlation analysis

**Figure 4:**
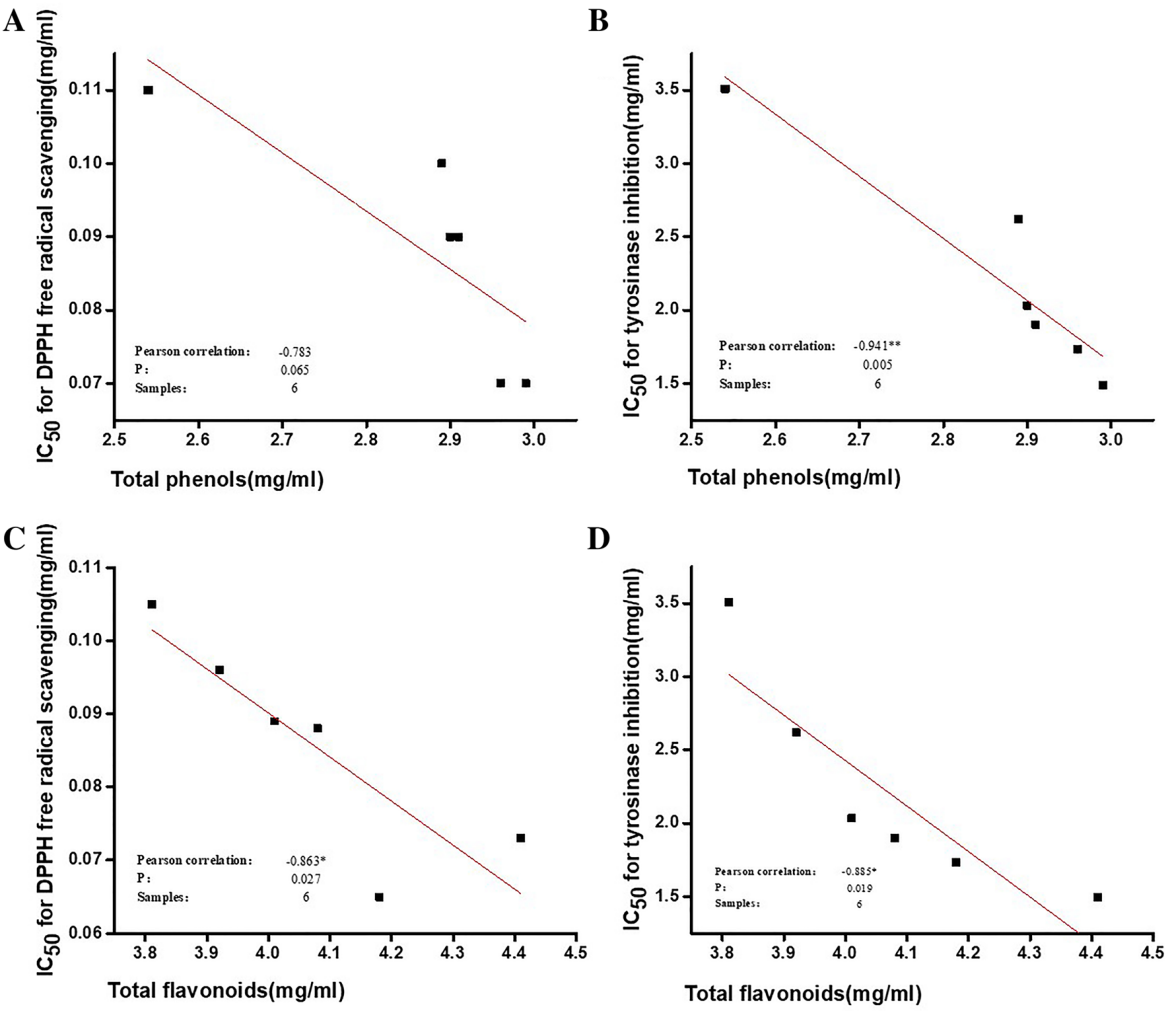
Correlation analysis. (**A**) DPPH radical scavenging activity and total phenolic content, (**B**) tyrosinase inhibition activity and total phenolic content, (**C**) DPPH radical scavenging activity and total flavonoid content, and (**D**) tyrosinase inhibition activity and total flavonoid content.

Our results revealed that an increase in total phenolic and flavonoid contents produced by increased fermentation time enhances the DPPH free radical scavenging rate and tyrosinase inhibition activity of FSR (Fig. 4). Pearson analysis demonstrated a moderate correlation between total phenolic content and DPPH free radical scavenging activity, but the difference was not statistically significant (*P* > 0.5) (Fig. 4A). Total phenolic contents were highly correlated with tyrosinase inhibition activity (*P* < 0.5) (Fig. 4B). These results indicate an increase in phenolic content after fermentation as an important factor triggering tyrosinase inhibition activity of FSR.

Pearson analysis showed that total flavonoid contents after fermentation were highly correlated with DPPH free radical scavenging rate and tyrosinase inhibitory activity (*P* < 0.5) (Fig. 4C,D). Moreover, the total flavonoid contents changed significantly with increases in fermentation time (Table 5).

#### Main effective compounds in the genus *Rosa* used by Bai people in Dali

**Figure 5:**
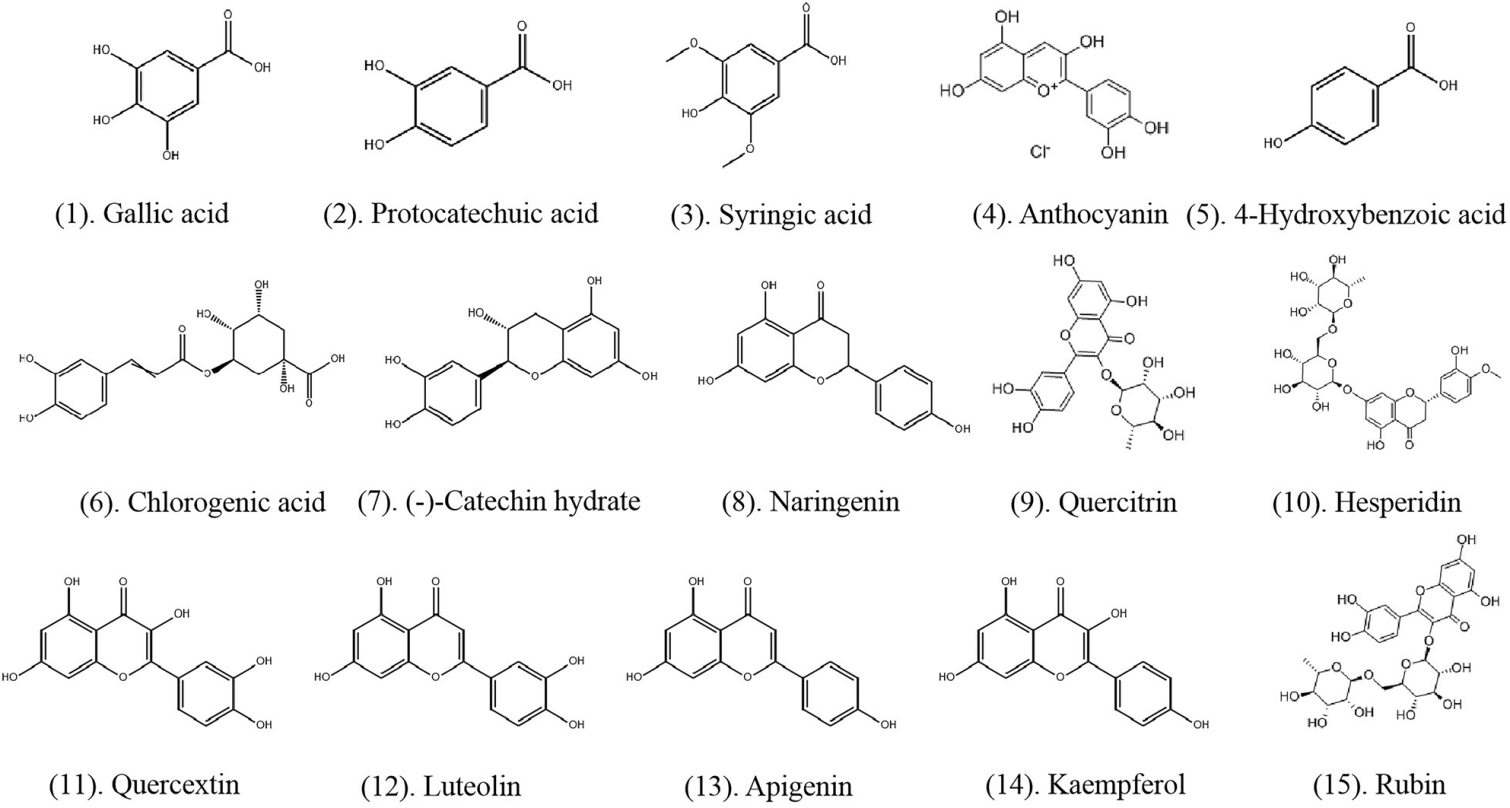
Structures of the main effective compounds in rose petals.

Petals from 15 plant species of the genus *Rosa* used in traditional applications in Dali Bai communities were collected; most were included in the previous phytochemical and pharmacological studies. The contents of the petals varied, but most of them contained flavonoids and phenols, which are known to be bioactive in vitro. Antioxidation is an important bioactivity of TFR in vitro. For example, according to Vinokur et al.^18^, the radical scavenging activity in rose petals is mostly due to the high content of phenolic compounds, especially free gallic acid (1; Fig. 5). protocatechuic acid (2), syringic acid (3), anthocyanin (4), 4-hydroxybenzoic acid (5), chlorogenic acid (6), and catechin hydrate (7) are also predominant phenols in rose petals^19^. These phenols have more hydroxyls, including o-dihydroxy, which demonstrate strong free radical scavenging and antioxidant abilities^20^. Flavonoids possess a galloyl ester in the C ring, which are important structures for chelation of metal ions, formation of complexes with metal ions, and inhibition of metal-initiated lipid oxidation. Therefore, flavonoids are able to effectively scavenge hydroxyl and peroxyl radicals^21–23^. The high concentration of flavonoids in *Rosa* resulted from the presence of a large amount of naringenin (8), with quercitrin (9), hesperidin (10), quercextin (11), luteolin (12), apigenin (13), and kaempferol (14) also comprising the main effective flavonoid compounds in rose petals; these flavonoid compounds contribute towards the antioxidant capacities of rose^19^. According to Jin^24^, the main flavonoids in rose petals include rubin (15), quercetin (11), kaempferol (14), and their derivatives; these compounds contributed more than 60% to the flavonoids in TFR rose petals, and our correlation analysis showed that they were significantly related to antioxidant activity. Flavonoids also slow tyrosinase activities by interacting with copper ions essential to the active site of tyrosinase. Tyrosinase, a key enzyme in skin pigmentation, catalyzes hydroxylation of monophenols to o-diphenols and oxidation of o-diphenols to o-quinones, which generates melanin^25^. Excessive tyrosinase can cause freckles, melasma, skin cancer, and age spots^26,27^. Therefore, flavonoids are considered to be a natural tyrosinase inhibitor.

## Discussion

Traditional edible flowers have been utilized as a therapeutic food in China for thousands of years^28^ because of their emollient, antibacterial, and anti-inflammatory properties. Many Dali Bai groups use specific plants because they are considered healthy, according to our ethnobotanical survey. Specific species, biochemical ingredients, or pharmacological qualities are the subject of most studies on the beneficial properties of food plants. Many researchers, for example, are looking for potential nutritional supplements against cancer in food plants. Antioxidants play a role in cancer prevention, and many types of research have been conducted on these substances. *Apium nodiflorum*, *Humulus lupulus*, *Silene vulgaris*^29^, *Nasturtium officinale*^30^, and *Leopoldia comosa* bulbs^31^ all have strong antioxidant capabilities. Our survey showed that *Rosa* 'Dianhong' petals are most commonly employed in traditional meals as a classic sauce, and their phenolic and flavonoid components have been linked to health benefits. Fermentation changes the chemical composition of food, potentially increasing its biological activity^32^. Fermentation is traditionally employed for food preservation^3,33^, but it is also an important biotransformation process for producing new products or crude materials^6,34^. Fermentation appears to increase the total phenolic and flavonoid content of FSR, as suggested by our findings. According to Pearson analysis, there is a strong link between total phenolic and flavonoid content in FSR and tyrosinase inhibition, consistent with prior research^35^. TFR can play a crucial role in conferring antioxidant and tyrosinase inhibitory action by boosting phenols and flavonoids. PDA isolated TFR-1, which was later recognized as belonging to *Saccharomyces rouxii*. It is a yeast commonly employed in soy sauce fermentation and safe to consume as it has been consumed for a long time in human history. This strain can withstand high osmotic pressure because it produces sugars, alcohols, acids, and other chemicals involved in sugar and energy metabolism to protect cells under hypertonic culture conditions^36^. Traditional *Rosa* fermentation uses a 50 percent sugar concentration to retain sweetness and taste under sugar stress to generate a high osmotic pressure environment that inhibits putrefactive, pathogenic, and other bacteria, most beneficial bacteria do not adapt to high osmotic pressure conditions, their usage is limited^37^. Only one strain, TFR-1, could tolerate the high osmotic pressure and play an important role in TFR processing in Dali Bai populations. Because of its excellent osmotic pressure resistance, non-toxicity to humans, and other possible functions in healthcare, the strain in this study is worth further investigation.

## Conclusion

In the Dali Bai villages in Northwest Yunnan, China, TFR is a traditional medical food. As evidenced by our ethnobotanical survey, local people sustain the traditional TFR process, which has been passed down from generation to generation through these activities. Our findings show that TFR prepared traditionally contains strain TFR-1 (*Saccharomyces rouxii*) as the most important microbial content that facilitates fermentation and impacts TFR quality. It increased the antioxidant activity and tyrosinase inhibitory activity of FSR. These critical functional activity alterations provide applicable research for TFR products, mainly cosmetic or nutraceutical products. Traditional medical food culture promotes environmental protection, protects fast vanishing traditional knowledge, and has numerous applications in other domains of human activity. To that end, this work underlines the importance of continuing research on traditional fermented food products and related traditional knowledge as a method of discovering new strains and expanding the commercial potential. Our findings also highlight the importance of traditional items in creating modern healthcare, food, and cosmetic businesses.

## Materials and methods

### Study area

This study was conducted in Dali Bai Autonomous Prefecture in northwest Yunnan Province, China. The average elevation of this area is 2090 m. The area receives an average of 776 mm annual precipitation, and the average annual temperature is 16.5 °C^38^. It is the homeland of the Bai people, and therefore Bai communities are most densely settled. Diverse geographical conditions mean Dali possesses rich flower resources, forming the necessary material for the inheritance of an edible flower culture. “Selling flowers by weighing” is an edible flower custom among 26 ethnic groups in Yunnan, where 140 species of edible flowers have been reported^39^. Dali Bai people’s “hundred flowers banquet” has a long history and is famous in Yunnan. *Rosa* species including *Rosa* ‘Dianhong’, *Rosa gallica* L., *Rosa banksiae* R. Br., and *Rosa multiflora* Thunb. var. Carnea Thory are among the common edible flowers used by the Bai people. These flowers are fried, steamed, boiled, pickled, and fermented prior to consumption. Fermentation ensures the availability of edible flower resources all year.

### Field work and ethnobotanical investigation

**Figure 6:**
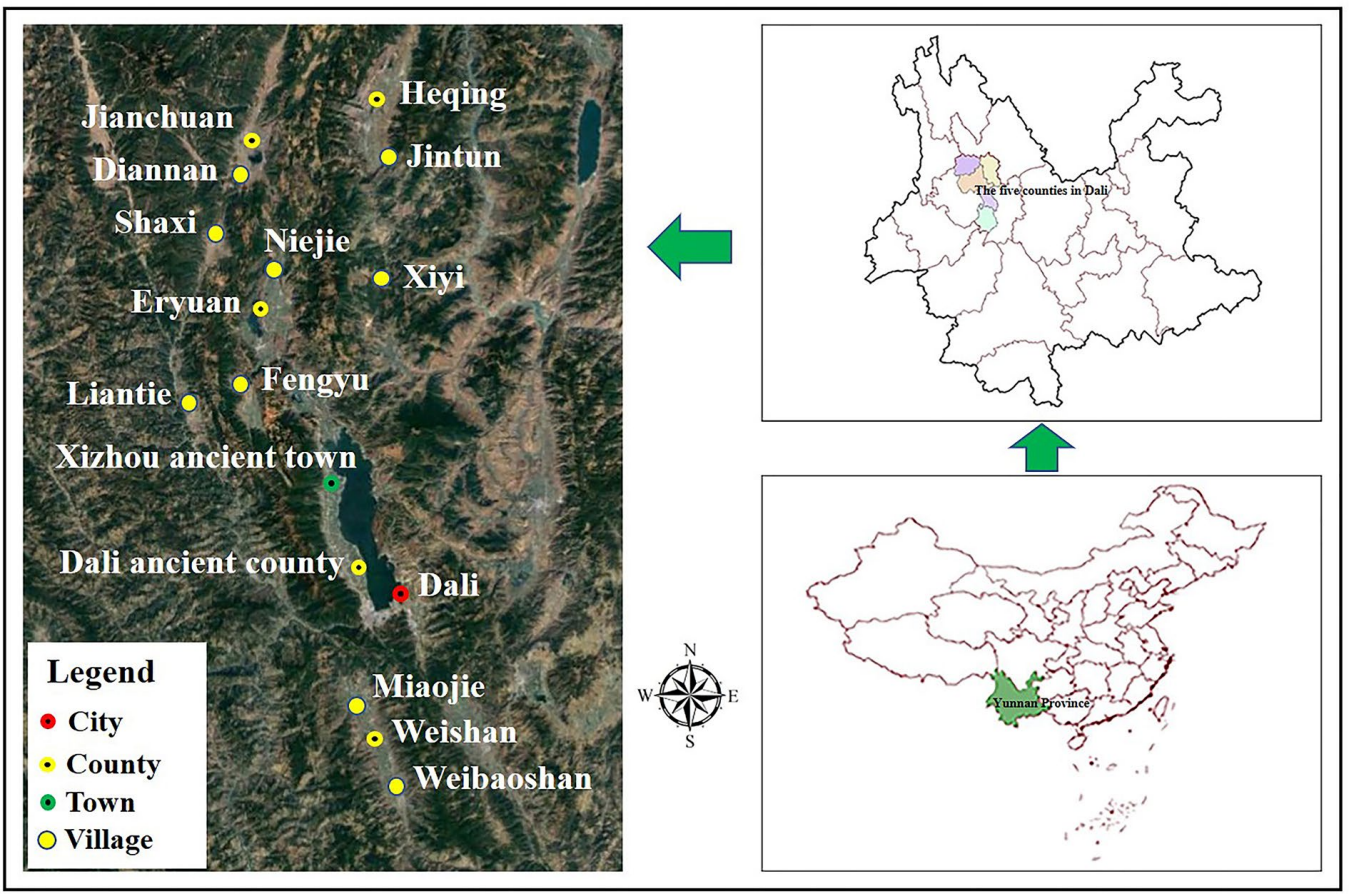
Location map of study sites. (Figure is created by ArcGIS 10.8, https://developers.arcgis.com, the satellite imagery was generated by Google Earth 7.3.4.8248, https://google-earth.en.softonic.com).

After consultation with local government officials and preliminary field visits, 15 traditional communities were selected for the investigation. The investigation was conducted between April 2017 and October 2018 in five counties: Dali, Weishan, Eryuan, Jianchuan, and Heqing (Fig. 6). Field work strictly obeyed the International Society of Ethnobiology Code of Ethics (2006 convention with 2008 additions)^40^. First, the purpose of the study was explained to key informants of Bai communities. Local community committees were then visited to obtain field study permission and request assistance. The assistance included introduction to community members and heirs of the intangible cultural heritage of TFR and organization of representative workshops. All field studies were carried out with informed consent. An ethnobotanical survey was conducted among 371 informants (167 men and 204 women) (Table 6) using the snowball sampling method to select potential informants^41^. Herbalists, farmers, merchants, and indigenous people were among the informants. They were engaged in the collection, production, sales, and use of the genus *Rosa.* Women are frequent users of the genus *Rosa* and accounted for 55% of informants*.* Men who are herbalists, farmers, and vendors are associated with the value chain of *Rosa*. All had long-term experience with the applications of the genus *Rosa*.

**Table 6:** Details of the informants.

Category	Subcategory	Number	Percent
Location	Dali	83	22.37
	Eryuan	76	20.49
	Weishan	65	17.52
	Jianchuan	69	18.60
	Heqing	78	21.02
Age (years)	26–35	82	22.10
	36–45	113	30.46
	46–55	109	29.38
	56–65	38	10.24
	66–75	21	5.66
	Over 75	8	2.16
Gender	Male	167	45
	Female	204	55

Semi-structured interviews were conducted with the consent of local people. The interviews focused on the following questions:

1. Does the community use the *Rosa* species? If yes, how many species are in traditional use?
2. Where do you get these plants?
3. How do you use these plants?
4. Does the community prepare TFR? If yes, which species of *Rosa* are in use?
5. Which ingredients are necessary for making TFR, and how do you prepare TFR?
6. How long does it require to make a TFR?
7. How do you consume TFR?
8. What are the benefits of TFR to health, and what effects have been noticed?

Voucher specimens of 20 *Rosa* species naturally occurring in the study area were collected. A taxonomist and ethnobotanist at Kunming Institute of Botany identified the voucher specimens. All specimens collected during the field survey were deposited in the Herbarium of Kunming Institute of Botany.

### Isolation, purification, and identification of strains

TFR prepared traditionally by the Bai people in Dali was used for strain isolation. TFR solution was prepared in sterile water at different concentrations (10^–1^–10^–5^). The dilution coating method was applied^42^. The diluent was inoculated onto a PDA (potato dextrose agar) medium for culture of fungi^43^ and onto NA (nutrient agar) medium for culture of bacteria^44,45^. After growth, fungal and bacterial colonies were purified using the plate streak method^45^. Purified materials, which were similar to yeast, were stored at 4 °C for preservation. PDA and NA medium were both from Qingdao Rishui Biotechnology Co., Ltd. The strain was named TFR-1 and deposited at the Kunming Institute of Botany, Chinese Academy of Sciences.

To ascertain the identity of strain TFR-1, its total genomic DNA was extracted using a DNA extraction kit from Beijing Tsingke Xinye Biotechnology Co., Ltd. (Tsingke) using the CTAB/SDS method^46^. The ITS1 region from the DNA sample was amplified by PCR using an Applied Biosystems 2720 thermal cycler, while fungal sequences were amplified using universal primers (Tsingke). Amplified PCR fragments were sequenced by Tsingke and used as a query sequence in a BLASTN search against the NCBI public database, following the method described by Blanc et al.^47^.

### Preparation of *Rosa* fermentation and sampling

TFR was prepared in the laboratory following the traditional method. Petals of *Rosa* ‘Dianhong’ that is traditionally used in TFR preparation in Dali were purchased, and 0.5 kg of petals was cut into small pieces to maximize extraction. Brown sugar (0.5 kg) was added and the mixture was kneaded manually until fully wet. The mixture was then placed into a 1-l fermentation bottle and 500 ml sterile water was added. Three bottles of this solution were prepared as replicates. The bottles were exposed to ultraviolet light for 45 min for sterilization on a super purgative working table. After 24 h, 10 ml *Rosa* ‘Dianhong’ solution from each bottle was removed and stored as sample FSR-0. Then, TFR-1 solution (15 ml) with a concentration of 4.15 × 10^6^ CFU/ml was added to the fermentation bottles, which were sealed with caps and stored at 28 °C in a thermostatic incubator. Fermentation solution of *Rosa* ‘Dianhong’ (FSR) from each bottle (10 ml) was sampled on days 3, 7, 14, 21, and 30 and labeled FSR-3, FSR-7, FSR-14, FSR-21, and FSR-30, respectively. Each of the samples was centrifuged at 4000 rpm for 15 min, and the supernatant liquid was stored at − 20 °C to cease further fermentation.

### Determination of total phenolic contents

Total phenolic contents of FSR were determined spectrophotometrically using Folin–Ciocalteu’s method as described by Kang et al.^48^ with slight modification. Diluted FSR (0.4%, v/v) was used in the analysis. A 10-μl aliquot of FSR was mixed with 0.25 ml 1 N Folin–Ciocalteu’s reagent in a 5-ml test tube. The mixture was covered and kept still for 2 min in the dark before 0.5 ml 12% (w/v) aqueous solution of Na_2_CO_3_ and 1.24 ml distilled water were added. The mixture was incubated for 1 h at room temperature and then ultraviolet absorbance was measured at 765 nm using a UV-5500PC (METASH) against a control. The control sample contained the same amount of chemicals with the FSR replaced by distilled water. Gallic acid (GA) was used as a standard for preparing a calibration curve. Total phenolic contents were expressed as mg GA equivalents per FSR.

### Determination of total flavonoid contents

Total flavonoid contents of FSR were measured using the aluminum chloride colorimetric assay^49^ with slight modification. Diluted FSR (concentration at 1.6%, v/v) was used in the analysis. A 40-μl aliquot of FSR was added into a 5-ml test tube containing 1.31 ml of distilled water and 75 μl of 5% (w/v) NaNO_2_ was then added. After 5 min, 75 μl 10% (w/v) AlCl_3_ was added and allowed to react for 6 min before 1 ml of 4% (w/v) NaOH dissolved in distilled water was added. The solution was mixed and kept for 12 min at room temperature before ultraviolet absorbance was measured against the control at 510 nm using a UV-5500PC (METASH). Control samples contained the same amount of chemicals except the FSR was replaced with distilled water. Rutin was used as a standard for constructing a calibration curve. Total flavonoid contents were expressed as mg rutin equivalents per of FSR.

### Free radical scavenging activity

The free radical scavenging activity of FSR was examined in vitro using DPPH (2, 2-diphenyl-1 picrylhydrazyl) radical as described by El Atki et al.^49^. Different concentrations of FSR or GA were added to ethanolic solution and mixed with DPPH dissolved in ethanolic solution so that the final concentration of DPPH was 0.1 mmol. The absorbance of the mixture was measured using a UV-5500PC (METASH) at 517 nm after 30 min of incubation at room temperature in darkness. A control mixture in which FSR was replaced by the equivalent amount of ethanolic solution was prepared following the same procedures. The percentage of inhibition was calculated using the following equation:

$$ \% {\text{DPPH}}\;{\text{scavenging}}\;{\text{activity}} = \left( {1 - {\text{OD}}_{{{\text{sample}}}} /{\text{OD}}_{{{\text{control}}}} } \right) \times 100 $$

Here, OD_control_ is the absorbance of the negative control and OD_sample_ is the absorbance of the sample. GA served as positive control. IC_50_ values were calculated as the concentration of causing a 50% inhibition of DPPH radical.

### Tyrosinase inhibition activity

Tyrosinase inhibitory activity was examined in vitro as described by Elena et al.^50^. Different concentrations of FSR or kojic acid (KA) were mixed with tyrosinase dissolved in 0.2 M phosphate buffer (pH 6.8) and kept still for 15 min at 37 °C. Next, 12.5 mM L-Dopa dissolved in 0.2 M phosphate buffer (pH 6.8) was added, so that the final concentration of tyrosinase was 25 U/ml and that of L-Dopa was 1.25 mM in the solution. Absorbance at 475 nm was measured using a UV-5500PC (METASH) after incubation for 5 min at room temperature. FSR was replaced by the equivalent amount of phosphate buffer in the control mixture prepared following the same procedures. The percentage of inhibition was calculated using the following equation:

$$ \% {\text{Tyrosinase}}\;{\text{inhibitory}}\;{\text{activity}} = \left( {1 - {\text{OD}}_{{{\text{sample}}}} /{\text{OD}}_{{{\text{control}}}} } \right) \times 100 $$

OD_control_ is the absorbance of the negative control and OD_sample_ is the absorbance of the sample. KA served as a positive control. IC_50_ values were calculated as the concentration of causing a 50% inhibition of tyrosinase.

### Statistical analysis

SPSS 22 (Statistical Product and Service Solutions, https://www.ibm.com/products/spss-statistics) was used for statistical analysis. Probit regression analysis was used to analyze the IC_50_ of DPPH free radical and tyrosinase inhibition. Pearson correlation analysis was used to determine the correlation of total phenolic and flavonoid contents with DPPH free radical scavenging activity and tyrosinase inhibition activity. Differences between means were determined using the least significant difference test at *P* < 0.05, and figures were drawn using OriginPro 2017 (https://www.originlab.com).

### Ethics approval and consent to participate

The Authors confirm that no animal/human studies have been carried out in the present. This study was part of a wider project entitled “Study on traditional fermented rose in Dali Bai Nationality”. We conducted this research in accordance with International Society of Ethnobiology (2006), ISE Code of Ethics (with 2008 additions), and the protocol was approved by Kunming Institute of Botany, Chinese Academy of Sciences (KIB) ethics committee (Supporting documents S1) and Center of Biodiversity and Indigenous Knowledge(CBIK) ethics committee (Supporting documents S2). Before data collection, we described the goals of this research to local informants and asked them to sign a Free andInformed Consent Term. We were authorized to collect plant specimens by Forestry and grassland Bureau of Dali Bai Autonomous Prefecture.

### Consent for publication

This manuscript does not contain any individual person's data, not applicable.

## Data Availability

All data, materials, and information are collected from the study sites.

## Abbreviations

TFR: Traditional fermented *Rosa*
FSR: Fermentation solution of *R**osa* ‘Dianhong’
TFC: Total flavonoid contents
TPC: Total phenolic contents
PDA: Potato dextrose agar (Medium)
NA: Nutrient agar (Medium)
DPPH: 2, 2-Diphenyl-1 picrylhydrazyl
GAE: Gallic acid equivalents
RE: Rutin equivalents
KA: Kojic acid
